# 6,7,8,9,10,11,12,13-Octa­hydro-5*H*-1,3-dithiole[4,5-*b*][1,4]dithia­cyclo­tridecine-2-thione

**DOI:** 10.1107/S1600536807068833

**Published:** 2008-01-09

**Authors:** Felora Heshmatpour, Fatemeh Darviche, Zeynab Emdadi, Bernhard Neumueller

**Affiliations:** a Chemistry Department, K. N. Toosi University of Technology, PO Box 15875-4416, Tehran 15418, Iran; b Fachbereich Chemie der Universität Marburg, Marburg, Germany

## Abstract

In the crystal structure of the title compound, C_12_H_18_S_5_, no significant inter­molecular π–π inter­actions are found. Weak inter­molecular C—S⋯π [S⋯centroid = 3.787 (1) Å] inter­actions and van der Waals forces may be effective in the stabilization of the structure.

## Related literature

For general background, see: Ferraris *et al.* (1973 ▶); Williams *et al.* (1992 ▶); Bechgaard *et al.* (1975 ▶); Engler *et al.* (1977 ▶); Kini *et al.* (1999 ▶); Li *et al.* (2000 ▶); Svenstrup & Becher (1995 ▶). For related literature, see: Kumar *et al.* (1998 ▶). For bond-length data, see: Allen *et al.* (1987 ▶).
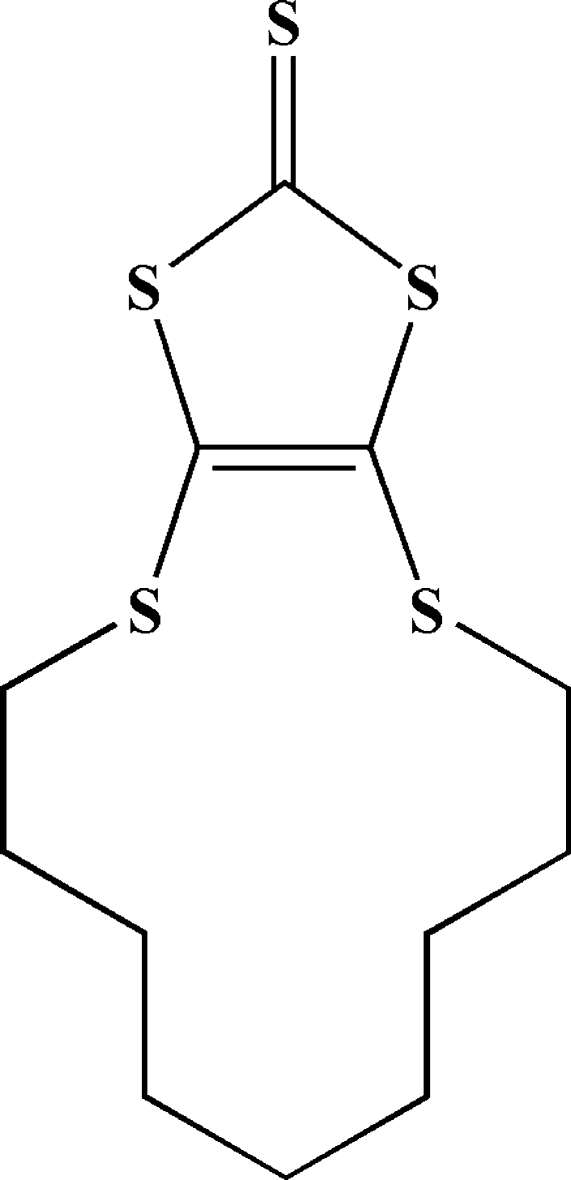


## Experimental

### 

#### Crystal data

C_12_H_18_S_5_
*M* *_r_* = 322.56
Monoclinic, 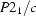

*a* = 5.588 (1) Å
*b* = 13.067 (1) Å
*c* = 20.446 (2) Å
β = 97.07 (1)°
*V* = 1481.6 (3) Å^3^
*Z* = 4
Mo *K*α radiation
μ = 0.76 mm^−1^
*T* = 173 (2) K
0.2 × 0.18 × 0.07 mm

#### Data collection

Stoe IPDS-II diffractometer
Absorption correction: numerical (shape of crystal determined optically; *X-RED32* and *X-SHAPE*; Stoe & Cie, 2005 ▶) *T*_min_ = 0.856, *T*_max_ = 0.948
20411 measured reflections
2866 independent reflections
1423 reflections with *I* > 2σ(*I*)
*R*_int_ = 0.107

#### Refinement

*R*[*F* ^2^ > 2σ(*F* ^2^)] = 0.031
*wR*(*F* ^2^) = 0.046
*S* = 0.90
2866 reflections
155 parameters
H-atom paramteres constrained
Δρ_max_ = 0.20 e Å^−3^
Δρ_min_ = −0.20 e Å^−3^

### 

Data collection: *X-AREA* (Stoe & Cie, 2005 ▶); cell refinement: *X-AREA*; data reduction: *X-RED32* (Stoe & Cie, 2005 ▶); program(s) used to solve structure: *SHELXS97* (Sheldrick, 2008 ▶); program(s) used to refine structure: *SHELXL97* (Sheldrick, 2008 ▶); molecular graphics: *ORTEP-3 for Windows* (Farrugia, 1997 ▶); software used to prepare material for publication: *WinGX* publication routines (Farrugia, 1999 ▶).

## Supplementary Material

Crystal structure: contains datablocks global, I. DOI: 10.1107/S1600536807068833/hk2410sup1.cif

Structure factors: contains datablocks I. DOI: 10.1107/S1600536807068833/hk2410Isup2.hkl

Additional supplementary materials:  crystallographic information; 3D view; checkCIF report

## Figures and Tables

**Fig. 1.**
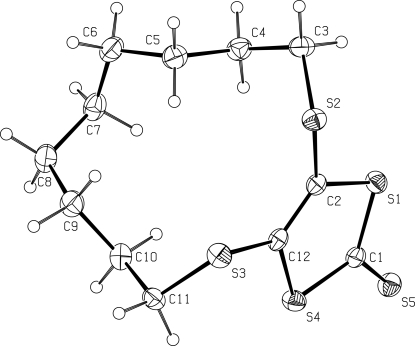
The molecular structure of the title molecule, with the atom-numbering scheme. Displacement ellipsoids are drawn at the 30% probability level.

## supplementary crystallographic information

### Comment

Since the discovery of the first organic metal TTF-TCNQ (TTF: tetrathiafulvalene TCNQ: 7,7,8,8-tetracyanoquinodimethane) (Ferraris *et al.*, 1973) organic electron donors with a TTF backbone have been widely investigated in terms of synthetic and structural as well as physical aspects (Williams *et al.*, 1992). The most conventional route to these electron donors is based on the coupling of 1,3-thiole-2-thione (one) derivatives promoted by trialkyl phosphite (Bechgaard *et al.*, 1975; Engler *et al.*, 1977; Kini *et al.*, 1999; Li *et al.*, 2000). Thus, the key precursors to these TTF-based electron donors are 1,3-thiole-2-thione (one) derivatives. Among them, 4,5-bisalkylthio-1,3-dithiole-2-thione can be routinely prepared by the reaction between a zinc complex of 1,3-dithiole-2 -thione-4,5-dithiolate or the anion 1,3-dithiole-2-thione-4,5-dithiolate generated *in situ* and suitable electrophilic reagents (Svenstrup & Becher, 1995). Thus the interest in the synthesis of various 1,3-dithiole-2-chalcogenone is evident and promoted us to take up this project. In continuation of our work in this field, we report herein the crystal structure of title ligand, (I).

In the molecule of (I) (Fig. 1), the bond lengths are within normal ranges (Allen *et al.*, 1987).

In the crystal structure, no significant intermolecular π–π interactions are observed. Weak intermolecular C—S···π interactions, with S1···*Cg*1 = 3.787 (1) Å [*Cg*1 denotes centroid of cyclotridecine ring; (S1/S4/C1/C2/C12), symmetry code: -1 + *x*, *y*, *z*] and van der Waals forces stabilize the crystal structure.

### Experimental

The synthesis of (I) was carried out *via* the coupling of 1,9-dibromooctane (1 mmol) with the zinc complex of 1,3-dithiole-2-thione-4,5-dithiolate (0.5 mmol) in acetone (5 ml) at 293 K. The color of the mixture was turned from red to yellow. The pure compound was obtained in 32% yield by washing of the crude product with chloroform, in which it is highly soluble (Kumar *et al.*, 1998).

### Refinement

H atoms were positioned geometrically, with C—H = 0.99 Å for methylene H, and constrained to ride on their parent atoms, with *U*_iso_(H) = 0.050 (2) Å^2^.

### Crystal data

**Table:**

C_12_H_18_S_5_	*F*_000_ = 680
*M**_r_* = 322.56	*D*_x_ = 1.446 Mg m^−^^3^
Monoclinic, *P*2_1_/*c*	Mo *K*α radiation λ = 0.71073 Å
Hall symbol: -P 2ybc	Cell parameters from 10000 reflections
*a* = 5.588 (1) Å	θ = 1.9–25.9º
*b* = 13.067 (1) Å	µ = 0.76 mm^−^^1^
*c* = 20.446 (2) Å	*T* = 173 (2) K
β = 97.07 (1)º	Plates, yellow
*V* = 1481.6 (3) Å^3^	0.2 × 0.18 × 0.07 mm
*Z* = 4	

### Data collection

**Table:**

Stoe IPDS-II diffractometer	*R*_int_ = 0.107
φ scans	θ_max_ = 25.9º
Absorption correction: numerical(shape of crystal determined optically; X-RED32 and X-SHAPE; Stoe & Cie, 2005)	θ_min_ = 1.9º
*T*_min_ = 0.856, *T*_max_ = 0.948	*h* = −6→6
20411 measured reflections	*k* = −15→16
2866 independent reflections	*l* = −25→25
1423 reflections with *I* > 2σ(*I*)	

### Refinement

**Table:**

Refinement on *F*^2^	Secondary atom site location: difference Fourier map
Least-squares matrix: full	Hydrogen site location: inferred from neighbouring sites
*R*[*F*^2^ > 2σ(*F*^2^)] = 0.031	H-atom parameters constrained
*wR*(*F*^2^) = 0.046	*w* = 1/[σ^2^(*F*_o_^2^) + (0.011*P*)^2^*P*] where *P* = (*F*_o_^2^ + 2*F*_c_^2^)/3
*S* = 0.90	(Δ/σ)_max_ = 0.002
2866 reflections	Δρ_max_ = 0.20 e Å^−^^3^
155 parameters	Δρ_min_ = −0.20 e Å^−^^3^
Primary atom site location: structure-invariant direct methods	Extinction correction: none

### Special details

**Table:**

Geometry. All e.s.d.'s (except the e.s.d. in the dihedral angle between two l.s. planes) are estimated using the full covariance matrix. The cell e.s.d.'s are taken into account individually in the estimation of e.s.d.'s in distances, angles and torsion angles; correlations between e.s.d.'s in cell parameters are only used when they are defined by crystal symmetry. An approximate (isotropic) treatment of cell e.s.d.'s is used for estimating e.s.d.'s involving l.s. planes.

### Fractional atomic coordinates and isotropic or equivalent isotropic displacement parameters (Å^2^)

**Table:**

	*x*	*y*	*z*	*U*_iso_*/*U*_eq_	
S1	0.15648 (13)	0.36424 (6)	0.20855 (4)	0.0339 (2)	
S2	0.60599 (13)	0.38230 (7)	0.30580 (4)	0.0359 (2)	
S3	0.55751 (13)	0.13199 (7)	0.32641 (4)	0.0384 (2)	
S4	0.11036 (13)	0.14671 (6)	0.22596 (4)	0.0356 (2)	
S5	−0.26751 (12)	0.25565 (8)	0.13696 (3)	0.03771 (19)	
C1	−0.0162 (4)	0.2563 (3)	0.18733 (11)	0.0301 (6)	
C2	0.3700 (5)	0.3076 (2)	0.26703 (13)	0.0292 (7)	
C3	0.4388 (5)	0.4824 (2)	0.34333 (15)	0.0403 (8)	
H31	0.5563	0.5299	0.3675	0.050 (2)*	
H32	0.3447	0.5219	0.3077	0.050 (2)*	
C4	0.2663 (6)	0.4446 (3)	0.39077 (15)	0.0405 (8)	
H41	0.1872	0.5045	0.4086	0.050 (2)*	
H42	0.1390	0.4023	0.3659	0.050 (2)*	
C5	0.3895 (5)	0.3822 (3)	0.44765 (14)	0.0438 (8)	
H51	0.5312	0.4206	0.4687	0.050 (2)*	
H52	0.4486	0.3175	0.4303	0.050 (2)*	
C6	0.2221 (5)	0.3571 (3)	0.50025 (14)	0.0458 (9)	
H61	0.3242	0.3340	0.5406	0.050 (2)*	
H62	0.1424	0.4214	0.5114	0.050 (2)*	
C7	0.0271 (5)	0.2770 (2)	0.48280 (14)	0.0434 (9)	
H71	−0.0170	0.2769	0.4344	0.050 (2)*	
H72	−0.1179	0.2972	0.5030	0.050 (2)*	
C8	0.0975 (6)	0.1682 (3)	0.50494 (14)	0.0470 (9)	
H81	0.1614	0.1704	0.5523	0.050 (2)*	
H82	−0.0507	0.1260	0.5008	0.050 (2)*	
C9	0.2818 (6)	0.1145 (3)	0.46824 (14)	0.0446 (9)	
H91	0.3430	0.0533	0.4936	0.050 (2)*	
H92	0.4199	0.1611	0.4653	0.050 (2)*	
C10	0.1780 (5)	0.0816 (2)	0.39871 (14)	0.0405 (8)	
H101	0.0509	0.0298	0.4021	0.050 (2)*	
H102	0.1009	0.1417	0.3753	0.050 (2)*	
C11	0.3638 (5)	0.0371 (2)	0.35761 (15)	0.0376 (8)	
H111	0.2777	−0.0008	0.3199	0.050 (2)*	
H112	0.4656	−0.0126	0.3850	0.050 (2)*	
C12	0.3500 (5)	0.2060 (2)	0.27516 (13)	0.0292 (7)	

### Atomic displacement parameters (Å^2^)

**Table:**

	*U*^11^	*U*^22^	*U*^33^	*U*^12^	*U*^13^	*U*^23^
S1	0.0339 (4)	0.0324 (5)	0.0350 (4)	−0.0027 (4)	0.0022 (3)	0.0027 (4)
S2	0.0295 (4)	0.0386 (5)	0.0396 (4)	−0.0053 (4)	0.0042 (4)	−0.0042 (4)
S3	0.0312 (4)	0.0394 (5)	0.0437 (5)	0.0050 (4)	0.0013 (4)	0.0023 (4)
S4	0.0350 (5)	0.0306 (5)	0.0399 (4)	−0.0020 (4)	−0.0002 (4)	−0.0017 (4)
S5	0.0349 (4)	0.0417 (5)	0.0353 (4)	−0.0011 (4)	−0.0006 (3)	−0.0007 (4)
C1	0.0328 (15)	0.0328 (17)	0.0268 (15)	−0.0002 (15)	0.0126 (11)	−0.0026 (15)
C2	0.0277 (18)	0.034 (2)	0.0275 (16)	0.0014 (13)	0.0094 (14)	−0.0018 (14)
C3	0.0429 (18)	0.035 (2)	0.0437 (19)	−0.0040 (15)	0.0080 (15)	−0.0085 (15)
C4	0.0424 (19)	0.039 (2)	0.0399 (18)	0.0058 (15)	0.0050 (15)	−0.0045 (16)
C5	0.0462 (18)	0.043 (2)	0.0419 (18)	−0.0003 (16)	0.0042 (14)	−0.0029 (16)
C6	0.055 (2)	0.050 (2)	0.0326 (17)	0.0093 (19)	0.0065 (15)	−0.0035 (17)
C7	0.0426 (17)	0.054 (3)	0.0351 (17)	0.0132 (16)	0.0122 (14)	0.0030 (16)
C8	0.054 (2)	0.051 (2)	0.0371 (18)	0.0110 (17)	0.0114 (15)	0.0080 (15)
C9	0.050 (2)	0.043 (2)	0.0398 (18)	0.0173 (17)	0.0005 (15)	0.0056 (16)
C10	0.0394 (18)	0.046 (2)	0.0360 (18)	−0.0001 (15)	0.0055 (14)	0.0061 (15)
C11	0.0439 (19)	0.0284 (19)	0.0400 (19)	0.0005 (15)	0.0027 (15)	0.0029 (14)
C12	0.0252 (17)	0.039 (2)	0.0244 (15)	0.0002 (14)	0.0058 (13)	−0.0016 (14)

### Geometric parameters (Å, °)

**Table:**

S1—C1	1.734 (3)	C5—H52	0.9900
S1—C2	1.746 (3)	C6—C7	1.521 (4)
S2—C2	1.750 (3)	C6—H61	0.9900
S2—C3	1.830 (3)	C6—H62	0.9900
S3—C12	1.754 (3)	C7—C8	1.528 (4)
S3—C11	1.813 (3)	C7—H71	0.9900
S4—C1	1.743 (3)	C7—H72	0.9900
S4—C12	1.753 (3)	C8—C9	1.519 (4)
S5—C1	1.636 (2)	C8—H81	0.9900
C2—C12	1.345 (3)	C8—H82	0.9900
C3—C4	1.531 (4)	C9—C10	1.529 (4)
C3—H31	0.9900	C9—H91	0.9900
C3—H32	0.9900	C9—H92	0.9900
C4—C5	1.515 (4)	C10—C11	1.529 (4)
C4—H41	0.9900	C10—H101	0.9900
C4—H42	0.9900	C10—H102	0.9900
C5—C6	1.545 (4)	C11—H111	0.9900
C5—H51	0.9900	C11—H112	0.9900
			
C1—S1—C2	97.99 (14)	C6—C7—C8	114.8 (3)
C2—S2—C3	101.15 (14)	C6—C7—H71	108.6
C12—S3—C11	101.98 (14)	C8—C7—H71	108.6
C1—S4—C12	97.81 (14)	C6—C7—H72	108.6
S5—C1—S1	124.7 (2)	C8—C7—H72	108.6
S5—C1—S4	123.4 (2)	H71—C7—H72	107.5
S1—C1—S4	111.88 (12)	C9—C8—C7	116.7 (2)
C12—C2—S1	116.3 (2)	C9—C8—H81	108.1
C12—C2—S2	124.3 (2)	C7—C8—H81	108.1
S1—C2—S2	119.16 (18)	C9—C8—H82	108.1
C4—C3—S2	115.4 (2)	C7—C8—H82	108.1
C4—C3—H31	108.4	H81—C8—H82	107.3
S2—C3—H31	108.4	C8—C9—C10	112.7 (3)
C4—C3—H32	108.4	C8—C9—H91	109.0
S2—C3—H32	108.4	C10—C9—H91	109.0
H31—C3—H32	107.5	C8—C9—H92	109.0
C5—C4—C3	113.5 (3)	C10—C9—H92	109.0
C5—C4—H41	108.9	H91—C9—H92	107.8
C3—C4—H41	108.9	C11—C10—C9	114.4 (2)
C5—C4—H42	108.9	C11—C10—H101	108.7
C3—C4—H42	108.9	C9—C10—H101	108.7
H41—C4—H42	107.7	C11—C10—H102	108.7
C4—C5—C6	113.1 (2)	C9—C10—H102	108.7
C4—C5—H51	109.0	H101—C10—H102	107.6
C6—C5—H51	109.0	C10—C11—S3	114.1 (2)
C4—C5—H52	109.0	C10—C11—H111	108.7
C6—C5—H52	109.0	S3—C11—H111	108.7
H51—C5—H52	107.8	C10—C11—H112	108.7
C7—C6—C5	117.4 (2)	S3—C11—H112	108.7
C7—C6—H61	107.9	H111—C11—H112	107.6
C5—C6—H61	107.9	C2—C12—S4	115.8 (2)
C7—C6—H62	107.9	C2—C12—S3	124.0 (2)
C5—C6—H62	107.9	S4—C12—S3	120.10 (19)
H61—C6—H62	107.2		
